# Cinnamon (*Cinnamomum* spp.): Phytochemical Constituents and Biological Potential from a Pharmacological Perspective

**DOI:** 10.3390/plants15203102

**Published:** 2026-10-10

**Authors:** Pratama Setiawan, Trivadila, Utami Dyah Syafitri, Muhammad Syukur, Wisnu Ananta Kusuma, Yanico Hadi Prayogo, Agus Saputra, Faridah Abas, Mohamad Rafi

**Affiliations:** 1 Department of Chemistry, Faculty of Mathematics and Natural Sciences, IPB University, Bogor 16680, Indonesia; prtmasetiawan@apps.ipb.ac.id (P.S.); trivadila@apps.ipb.ac.id (T.); 2 Tropical Biopharmaca Research Center, International Research Institute of Food, Nutrition, Health, and Halal, IPB University, Bogor 16128, Indonesia; utamids@apps.ipb.ac.id (U.D.S.); ananta@apps.ipb.ac.id (W.A.K.); yanicopryg@apps.ipb.ac.id (Y.H.P.); 3 Department of Computational Statistics, School of Data Science, Mathematics, and Informatics, IPB University, Bogor 16680, Indonesia; 4 Department of Agronomy and Horticulture, Faculty of Agriculture, IPB University, Bogor 16680, Indonesia; muhsyukur@apps.ipb.ac.id; 5 Division of Computational Intelligence and Optimization, School of Data Science, Mathematics, and Informatics, IPB University, Bogor 16680, Indonesia; 6 Department of Forest Products, Faculty of Forestry and Environment, IPB University, Bogor 16680, Indonesia; 7 Faculty of Medicine and Veterinary Medicine, Nusa Cendana University, Kupang 85001, Indonesia; saputra.a@staf.undana.ac.id; 8 Department of Food Science, Faculty of Food Science and Technology, Universiti Putra Malaysia, Serdang 43400, Selangor, Malaysia; faridah_abas@upm.edu.my

**Keywords:** bioactive compounds, *Cinnamomum*, natural products, pharmacological activity, phytochemicals

## Abstract

The genus *Cinnamomum* (Lauraceae) has a long history of use in traditional medicine and continues to attract scientific interest due to its diverse pharmacological properties. This review summarizes the ethnopharmacological uses of *Cinnamomum* species, including major investigated species such *as C. verum*, *C. cassia*, *C. burmannii*, *C. camphora*, and other reported species, plant parts utilized, phytochemical composition, biological activities, underlying mechanisms, and safety considerations. Evidence from the literature indicates that bark and leaves are the plant parts most frequently investigated. The phytochemical profile of *Cinnamomum* comprises diverse volatile and non-volatile constituents, including bioactive compounds such as cinnamaldehyde, eugenol, cinnamic acid, coumarin, and phenolic compounds, with composition influenced by species, geographical origin, plant part, and extraction method. Chemometric approaches have been applied to characterize metabolite variations and their association with biological activities. Preclinical studies demonstrate antioxidant, anti-inflammatory, antidiabetic, anticancer, antimicrobial, cardioprotective, and neuroprotective effects across several *Cinnamomum* species. These activities are associated with regulation of oxidative stress, NF-κB/AP-1-mediated inflammatory responses, PI3K/AKT-related metabolic regulation, and apoptosis-related mechanisms. Despite these promising findings, substantial variability in plant materials, extraction procedures, chemical standardization, experimental models, dose selection, and safety profiles across different *Cinnamomum* species limits comparison among studies and clinical translation. Further research integrating phytochemical characterization, metabolomics and chemometrics, standardized bioactivity assays, toxicological evaluation, and clinical studies is required to establish the efficacy, safety, and quality of *Cinnamomum* species.

## 1. Introduction

Natural products represent an important source of therapeutic agents due to their diverse chemical structures and biological activities. They contain various primary and secondary metabolites that contribute to medicinal applications and provide valuable resources for drug discovery [1,2,3]. Medicinal plants have demonstrated therapeutic roles in the treatment of various conditions at a relatively lower cost and with fewer side effects than conventional drugs [4]. There is enormous potential in natural products, but less than 10% of the world’s biodiversity has been evaluated for its biological activity. Biologically active compounds derived from plants are widely used in various applications, especially in the field of health [5]. The unique properties of each plant have long been a source of potential drug candidates, and many of them have become natural drug candidates [6].

One natural product classified as a medicinal plant is cinnamon, a tropical plant belonging to the genus *Cinnamomum* and the Lauraceae family. In addition to being used as a flavoring agent, this plant is also used for traditional medicine purposes [7]. Various species of cinnamon have been used in traditional medicine in Asia, the Middle East, North Africa, and Europe, especially to treat digestive disorders, infections, inflammation, and other metabolic disorders [8].

The increasing prevalence of disease poses significant challenges to global healthcare system and adversely affects quality of life [9]. Conventional therapy has limitations such as long-term side effects, high costs, and the emergence of chronic disease resistance. This encourages the exploration of alternative therapeutic agents derived from natural ingredients, which are considered safer, more affordable, and potentially effective at multiple levels [10,11,12]. The *Cinnamomum* genus occupies an important position in modern pharmacology due to its diverse bioactive compounds [13]. In response to the need, *Cinnamomum* species have attracted considerable attention in pharmacological research because they possess various biological activities that support human health, including antibacterial, antidiabetic, antioxidant, anti-inflammatory, antitumor, and neuroprotective activities [14,15,16]. Thus, cinnamon serves as a bridge between traditional phytotherapy and modern drug development, with the potential to address the challenges posed by rising disease prevalence more comprehensively and sustainably.

These biological activities are closely associated with the diverse phytochemical profiles of *Cinnamomum* species, including volatile compounds found in essential oils, such as monoterpenoids, sesquiterpenoids, alcohols, phenols, aldehydes, ketones, esters, and acids, as well as non-volatile compounds, including phenolic compounds, flavonoids, lignans, coumarins, tannins, phenylpropanoids, and other polyphenolic compounds [13,17,18,19,20,21]. In general, the relationship between phytochemical composition and biological activity suggests that the presence and interactions of bioactive components can enhance overall biological activity [22]. Therefore, phytochemical studies play an important role in elucidating the mechanism of action, assessing therapeutic potential, and providing a scientific basis for the pharmacological application of *Cinnamomum* species.

**Figure 1:**
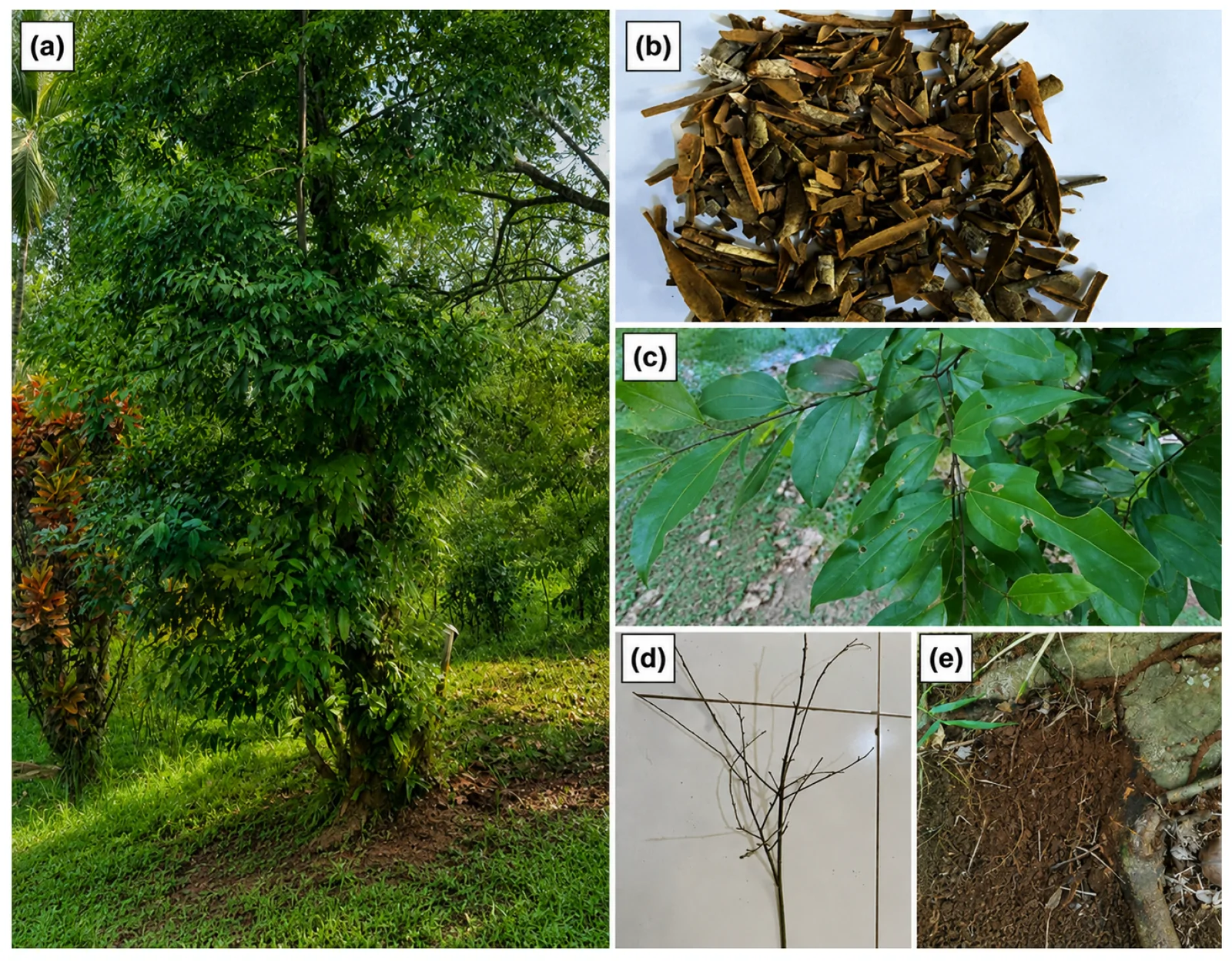
Morphological characteristics and different parts of *C. burmannii* plant. (**a**) Whole plant growth in Indonesia; (**b**) bark; (**c**) leaves; (**d**) twigs; and (**e**) roots.

Various studies have reported the phytochemical potential and biological activities of *Cinnamomum* species, but the available data remain fragmented and scattered across species, extract types, and methodological approaches. In addition, research often focuses on phytochemical characterization and biological activity evaluation, leaving the relationship between bioactive compounds and the plant’s pharmacological effects incompletely described. This condition limits the comprehensive understanding of the relationship between bioactive compounds and the biological effects of medicinal plants. Therefore, this review article aims to comprehensively examine the phytochemical content and biological activities of various *Cinnamomum* species from a pharmacological perspective. This review is expected to provide scientific information supporting the development of *Cinnamomum* species as candidates for natural medicine and future health research. Among the various *Cinnamomum* species, *C. burmannii* is one of the most widely utilized species, particularly as a spice and traditional medicinal plant. Its morphological characteristics and commonly used plant parts are illustrated in Figure 1.

## 2. Literature Review Methodology

Data for this review were collected through a comprehensive literature search in various scientific databases, including Google Scholar, PubMed, Scopus, and Web of Science. Journal platforms such as ScienceDirect, MDPI, and DOAJ were also used. The search was conducted to identify research and review articles relevant to plants from the genus *Cinnamomum*, cinnamon, medicinal plants, the therapeutic potential of plant-derived bioactive compounds, phytobioactive compounds, antioxidants, anti-inflammatory, antidiabetic, anticancer, antimicrobial, and related terms. Publications that extensively investigated the biological activities and therapeutic effects of bioactive compounds derived from *Cinnamomum* species were included in this review. Experimental approaches in vitro, in vivo, and in silico were also included. The selected articles were then qualitatively analyzed to provide a comprehensive picture of the relationship between phytochemical content and the pharmacological potential of *Cinnamomum* species.

## 3. Botanical Review and Diversity of Cinnamomum Species

The most commonly used parts of the plant are the bark and leaves [23,24,25,26]. Species from the genus *Cinnamomum* have been widely reported to possess significant pharmacological potential and have been extensively investigated in various experimental studies. Therapeutic differences within this genus are reflected in the variety of species, plant parts used, and their biological activities. A concise and systematic comparative overview of the most commonly used *Cinnamomum* species in pharmacological studies, along with their plant parts and primary biological activities, is presented in Table 1.

## 4. Phytochemical Content of Cinnamomum Species

The genus *Cinnamomum* contains diverse phytochemical constituents that contribute to its chemical characteristics and biological potential [55]. In general, these compounds can be grouped into volatile and non-volatile compounds [56]. Although they differ in physicochemical properties, distribution in plant tissues, and identification methods, these two groups of compounds contribute to the chemical characteristics and biological potential of cinnamon plants [14,46]. This phytochemical diversity reflects the complexity of secondary metabolism in *Cinnamomum* species and provides an important basis for understanding the pharmacological variations reported across various species and plant parts [3,55].

The phytochemical composition in *Cinnamomum* species is not uniform because it is influenced by several factors, including species, location, and the part of the plant analyzed [39]. Variations between species can result in significant differences in the types and concentrations of bioactive compounds [29,39,57]. Different extraction methods can affect the selectivity of the compounds obtained [58,59]. In addition, differences in plant parts and growing environmental conditions contribute to variations in the secondary metabolite profiles detected [39,60].

Identification and characterization of phytochemical compounds from *Cinnamomum* species are generally carried out using a combination of chromatographic and spectrometric methods to obtain comprehensive information on their chemical profiles. Volatile compounds are generally analyzed using gas chromatography–mass spectrometry (GC-MS) to separate components based on volatility and to determine tentative structures by matching mass spectra with available databases [56,61]. In addition, non-volatile compounds with higher polarity and more complex structures are mostly identified using high performance liquid chromatography coupled with diode array detector (HPLC-DAD) or liquid chromatography-mass spectrometry (LC-MS/MS), which can provide quantitative and qualitative information with high sensitivity [62,63]. This analytical approach is often complemented by spectroscopic techniques, such as Fourier-transform infrared spectroscopy (FTIR) or nuclear magnetic resonance (NMR), to confirm the structures of certain compounds, especially isolated compounds [56,64]. The use of appropriate identification methods is an important aspect for ensuring accurate determination of bioactive compounds and for supporting the interpretation of the relationship between phytochemical content and the pharmacological potential of *Cinnamomum* species.

The diversity of phytochemicals in *Cinnamomum* species indicates that this genus is a complex and highly potent source of secondary metabolites. The compounds reported include various chemical groups from different plant parts of each species, identified using different methods. A concise and systematic comparative overview of the reported phytochemical compounds in *Cinnamomum* species, along with their chemical classification and related botanical sources, is summarized in Table 2.

**Figure 2:**
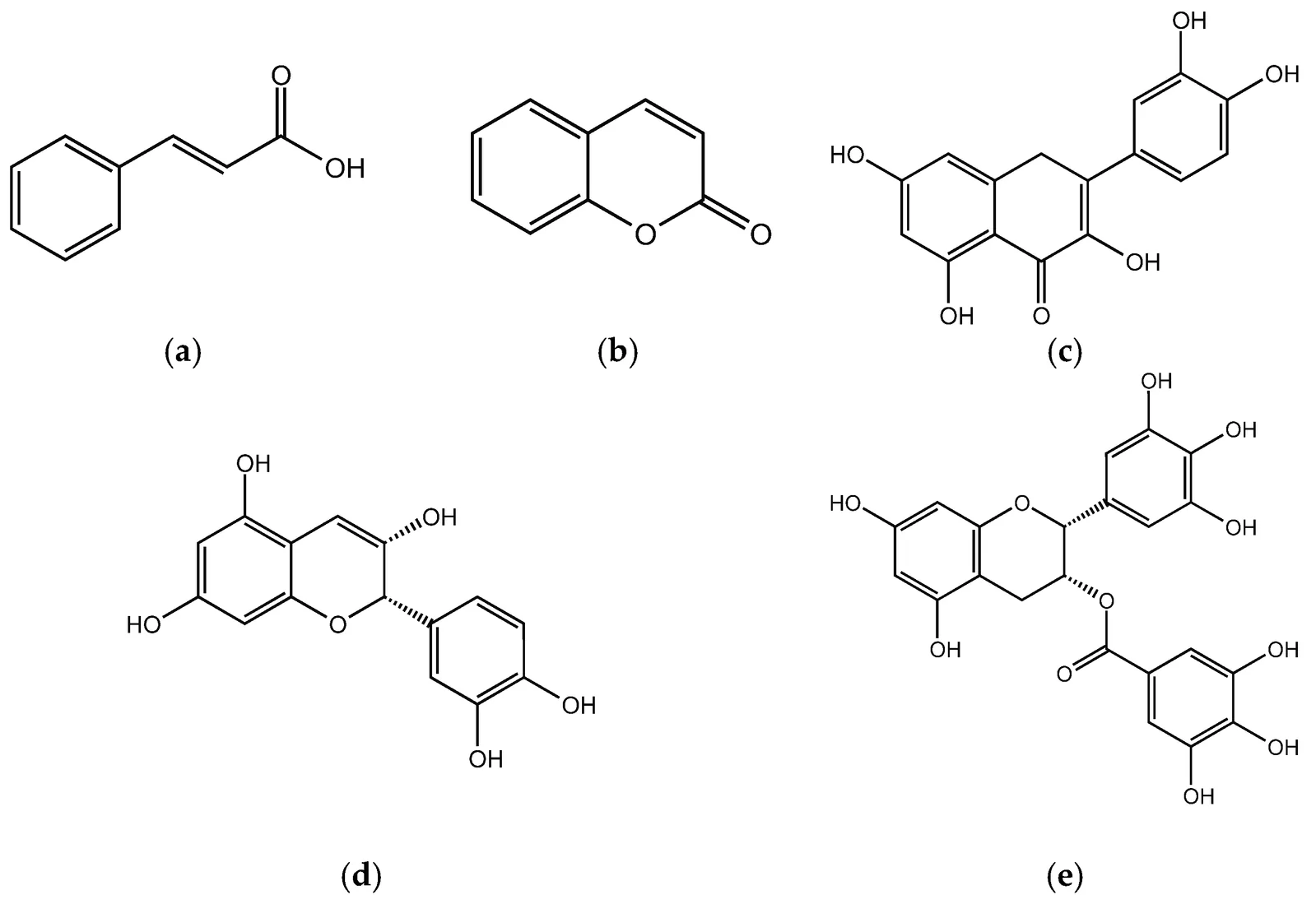
Representative chemical structures of major non-volatile constituents identified in *Cinnamomum* species. (**a**) Cinnamic acid, (**b**) coumarin, (**c**) quercetin, (**d**) epicatechin, and (**e**) epigallocatechin gallate.

In addition to volatile constituents, *Cinnamomum* species contain diverse non-volatile phytochemicals, including phenylpropanoids, flavonoids, phenolic acids, coumarin derivatives, and other polar secondary metabolites. Several representative compounds, such as trans-cinnamic acid, cinnamic acid, epicatechin, epigallocatechin gallate, quercetin, and coumarin, have been identified in different *Cinnamomum* species. These non-volatile constituents contribute to the biological potential of *Cinnamomum* through antioxidant, anti-inflammatory, and other pharmacological activities, although their distribution varies depending on species, plant part, geographical origin, and extraction method. Representative chemical structures of major non-volatile constituents identified in *Cinnamomum* species are shown in Figure 2.

## 5. Pharmacological Activity and Mechanism of Action of Cinnamomum Species

The pharmacological activity of *Cinnamomum* species has been widely evaluated using various research designs and experimental models, including in vitro, in vivo, and in silico tests [67,72]. These differences in testing approaches affect the parameters observed and the interpretation of the biological effectiveness of the samples tested, so study results cannot always be directly compared. Variations in sample types contribute to differences in reported biological responses [39]. Given this heterogeneity, this section summarizes the main pharmacological activities of *Cinnamomum* species based on experimental evidence, including antioxidant, anti-inflammatory, antidiabetic, antimicrobial, anticancer, cardioprotective, and neuroprotective effects.

### 5.1. Antioxidants

Oxidative stress occurs when the production of reactive oxygen species (ROS) exceeds the capacity of antioxidant defense systems, resulting in oxidative damage that may contribute to lipid peroxidation, endothelial dysfunction, and the progression of atherosclerosis [73]. ROS can originate from endogenous processes, including mitochondrial metabolism and inflammatory responses, as well as from exogenous factors such as radiation, ultraviolet exposure, air pollution, and tobacco smoke [74]. In this context, *Cinnamomum* species have gained attention as potential sources of natural antioxidants due to their diverse phenolic and other bioactive constituents.

The results of the antioxidant activity test showed that *Cinnamomum* species has significant antioxidant potential, which is influenced by the solvent type and concentration of the extraction solvent. The extract obtained with 80% ethanol showed the highest free radical-scavenging activity based on the DPPH test, as well as a stronger reducing power test when compared to other extracts (*p* < 0.05), which indicates the effectiveness of semipolar solvents in extracting antioxidant bioactive compounds. Meanwhile, the water-based extract exhibited greater metal-ion chelating ability, indicating a distinct antioxidant mechanism. The higher total phenolic content in the ethanol extract was positively correlated with increased antioxidant activity, thus confirming that *C. cassia* is a potential source of natural antioxidants and that extraction method influences its antioxidant activity [75].

In vivo testing showed that *C. cassia* leaf extract has significant antioxidant activity in a D-galactose-induced aging model. Oral administration of the extract can increase the activity of endogenous antioxidant enzymes, especially superoxide dismutase (SOD) and glutathione peroxidase (GSH-Px). In addition, the extract can significantly reduce malondialdehyde (MDA) levels as an indicator of lipid peroxidation. This increase in antioxidant status was consistently observed in serum, brain tissue, and liver, with a dose-dependent effect. These findings indicate that *C. cassia* extract suppresses oxidative stress by strengthening the endogenous antioxidant defense system and inhibiting oxidative damage to biomolecules [76].

At the compound level, metabolite profiling of *C. verum* leaf oil revealed a diverse composition of phenolic and volatile constituents that may collectively contribute to its antioxidant properties. LC-HRMS analysis identified several phenolic compounds, including quercetin, luteolin, apigenin, rutin, naringenin, and hispidulin, while GC-MS and GC-FID analyses showed that (E)-cinnamaldehyde was the predominant volatile constituent, accompanied by β-caryophyllene, trans-cinnamyl acetate, eugenol, linalool, and other minor components. The leaf oil also exhibited strong radical-scavenging activity in DPPH and ABTS assays, as well as considerable Fe^3+^, Cu^2+^, and Fe^3+^-TPTZ reducing capacities. Notably, (E)-cinnamaldehyde accounted for 72.98% of the volatile fraction, suggesting that this major constituent, together with phenolic compounds and other terpenoid components, may be associated with the antioxidant properties observed in *C. verum* leaf oil [5]. However, the contribution of individual constituents requires further confirmation using isolated compounds and mechanistic studies.

Additional evidence has been reported for *C. malabatrum* leaf essential oil, which exhibited dose-dependent antioxidant activity across several in vitro assays. The essential oil showed DPPH, ABTS, and H_2_O_2_ radical-scavenging activities with IC_50_ values of 21.50 ± 0.17, 36.91 ± 0.41, and 42.77 ± 0.34 µg/mL, respectively, together with ferric-reducing and lipid peroxidation-inhibitory activities. GC-MS analysis identified linalool as the major constituent, accompanied by cinnamaldehyde, caryophyllene, benzyl benzoate, eugenol, and humulene, suggesting that the observed antioxidant effect may reflect the combined contribution of these volatile constituents [51].

### 5.2. Anti-Inflammatory

Atherogenesis is a chronic inflammatory process in which, in the initial stage, there is recruitment of leukocytes and an increase in proinflammatory cytokines that play a significant role. Activation of the inflammatory cascade in vascular endothelial cells increases the biosynthesis of adhesion molecules, including vascular cell adhesion molecule-1 (VCAM-1), intracellular adhesion molecule-1 (ICAM-1), and E-selectin. In addition, it can also increase the expression of the chemokine monocyte chemoattractant protein-1 (MCP-1). This condition encourages the recruitment and retention of circulating monocytes into the intima layer, which then differentiate into macrophages, form foam cells, and worsen the development of atherosclerotic lesions through the release of various proinflammatory mediators [73].

In vitro testing results showed that *C. verum* extract has significant anti-inflammatory activity against stimulated inflammatory cell models. Treatment of the extract at non-cytotoxic concentrations significantly reduced the expression of various major inflammatory mediators, including proinflammatory cytokines (such as IL-1β and IL-8), chemokines, and the inflammatory enzyme cyclooxygenase-2 (COX-2). This anti-inflammatory effect is related to the ability of *C. verum* to suppress intracellular ROS production by inhibiting NADPH oxidase expression, thereby reducing oxidative stress, which amplifies inflammatory responses. In addition, this extract has been shown to inhibit the activation of inflammatory signaling pathways by suppressing the phosphorylation of related protein kinases and inhibiting the activation and nuclear translocation of proinflammatory transcription factors such as NF-κB and AP-1. This confirms that the anti-inflammatory activity of *C. verum* is mediated by inhibition of oxidative stress and by regulation of major anti-inflammatory signaling pathways at the molecular level. Furthermore, compound-level analysis identified cinnamic acid and coumarin as constituents of *C. verum* that have demonstrated anti-inflammatory activity when evaluated individually. Both compounds suppressed the expression of proinflammatory mediators and were associated with inhibition of PKCδ membrane translocation and downregulation of NOX2 expression. These findings suggest that these compounds may possess anti-inflammatory properties. However, their specific contribution to the activity observed in the whole extract remains to be fully established [27].

The modulation of the inflammatory response by *C. verum* is also reflected in its ability to maintain cellular function under inflammatory conditions by protecting against the disruption of cell integrity and suppressing the excessive secretion of inflammatory mediators. In cell models exposed to proinflammatory stimuli, treatment with *C. verum* extract significantly reduced increased cell permeability and suppressed the release of inflammatory cytokines into the extracellular environment. These effects indicate that the extract not only acts at the cellular level but also contributes to the functional regulation of the inflammatory process. Thus, *C. verum* has the potential to act as an inflammatory modulating agent that works by controlling key mediators and stabilizing cellular responses to chronic inflammatory stimuli [77].

### 5.3. Antidiabetic

Diabetes mellitus (DM) is a metabolic disorder characterized by persistent hyperglycemia resulting from impaired insulin secretion, insulin action, or both [78]. Diabetes is divided into three types: type 1 diabetes mellitus (DM 1), type 2 diabetes mellitus (DM 2), and gestational diabetes mellitus. The causes and complications of DM 2 can be managed effectively compared to type 1 and gestational diabetes. Diabetes mellitus can be controlled with a healthy lifestyle and appropriate dietary choices. In addition, the use of medicinal plants is another effective approach to controlling DM 2 due to their phytochemical content, including phenolics, flavonoids, anthocyanins, carotenoids, terpenoids, and others [79].

The test results showed that cinnamon exerted significant antidiabetic effects in a type 2 diabetes model induced by a high-fat diet and streptozotocin. Oral administration of the extract reduced fasting blood glucose levels, improved glucose tolerance, decreased the insulin resistance index (HOMA-IR), and led to improvements in serum lipid profiles. Mechanistic analysis showed that these effects were mediated by activation of the PI3K/AKT signaling pathway in liver tissue and the FXR/FGF15 axis in the intestine, which plays an important role in regulating glucose and lipid homeostasis. In addition, cinnamon extract modulated the composition of the gut microbiota and related metabolites, which collectively contributed to increased insulin sensitivity and improved metabolic disorders in type 2 diabetes [80].

Based on in vitro and in silico approaches, *C. verum* shows antidiabetic potential through glycemic control at two main levels, namely carbohydrate digestion and glucose utilization by peripheral tissues. Ethanolic bark extract of *C. verum* has been reported to significantly inhibit α-glucosidase and α-amylase activities, which help slow carbohydrate hydrolysis and suppress postprandial glucose spikes. In addition, increased glucose uptake in differentiated adipocytes indicates modulation of insulin-target-cell function. Further molecular analysis indicates that the main phenolic compounds in *C. verum* may interact with GLUT4 and PPAR-γ, two key proteins involved in glucose transport and insulin sensitivity. This interaction pattern suggests a possible molecular basis underlying the antidiabetic potential of *C. verum*. However, these in silico predictions require further experimental validation to confirm their biological relevance. More specifically, HPLC analysis identified gallic acid, quercetin, vanillic acid, and ferulic acid as the principal phenolic constituents present in C. verum extract and potentially associated with its antidiabetic activity, with quercetin showing the strongest predicted binding affinity among the identified compounds [81].

Beyond these experimental findings, cinnamon has also demonstrated antidiabetic potential through multiple biological mechanisms reported in previous in vitro and in vivo studies. Cinnamon contains various bioactive compounds, including cinnamaldehyde, polyphenols, flavonoids, tannins, and cinnamic acid, which contribute to glucose regulation by inhibiting carbohydrate-digesting enzymes and improving insulin sensitivity. Several studies have shown that cinnamon extracts can inhibit α-amylase and α-glucosidase activities, thereby delaying carbohydrate hydrolysis and reducing postprandial glucose elevation. Furthermore, cinnamon-derived compounds may enhance glucose uptake through GLUT4 regulation and improve insulin signaling pathways, suggesting its potential role as a complementary approach in diabetes management [82].

### 5.4. Anticancer and Chemopreventive

Cancer is a general term for a diverse group of diseases characterized by the uncontrolled growth and proliferation of abnormal cells, which can occur in almost all tissues of the body during the life cycle of living organisms [83]. Deregulation of cellular signaling pathways plays a fundamental role in cancer pathogenesis by modulating processes such as initiation, metastasis, drug resistance, apoptosis evasion, and genetic and epigenetic changes [84]. Cancer may be prevented, at least in part, by delaying its initiation and progression through exposure to natural compounds found in food. Most of these compounds are derived from plants and are generally classified as phytochemicals. Various groups of phytochemicals, including polyphenols, flavonoids, terpenoids, saponins, anthocyanins, phytosterols, isothiocyanates, and organosulfur compounds, have different structural characteristics. Despite these structural differences, many of them have significant chemopreventive and chemotherapeutic effects against various types of cancer [85].

Analysis of *C. verum* showed strong anticancer and chemopreventive activity in an in vivo breast carcinoma model and was confirmed through mechanistic evaluation. In a mouse model of induced mammary carcinomagenesis, feeding significantly reduced tumor incidence (−15.5%) and improved histopathological characteristics by reducing the ratio of high-grade carcinoma relative to controls. The chemopreventive effect was accompanied by changes in molecular biomarkers consistent with inhibition of tumor progression, namely increased apoptosis markers (increased expression of caspase-3 and Bax) and decreased proliferation markers (Ki-67), angiogenesis (VEGF), and CD24 expression. In addition, in a syngeneic 4T1 mouse model, high-dose treatment reduced tumor volume by 44% and decreased the mitotic index in a dose-independent manner, indicating direct suppression of cancer cell proliferation [67].

*C. tamala* essential oil through an integrative approach combining chemical profiling, molecular target analysis, and biological validation in a lung cancer model. The GC-MS-profiled essential oil contained various bioactive constituents, and several selected compounds were predicted to interact with key targets implicated in non-small-cell lung cancer (NSCLC). Network analysis identified several key genes as hubs. including JUN, P53, IL6, MAPK3, HIF1A, and CASP3. In addition, cinnamaldehyde and ethyl cinnamate were identified as major compounds potentially involved in the observed activity based on chemical profiling and computational analysis. However, their individual anticancer effects require further experimental validation. Pathway enrichment indicates the involvement of apoptotic mechanisms, as well as inflammatory and proliferative signaling pathways, including TNF, IL-17, and MAPK signaling, which are relevant to cancer progression. In vitro validation also shows that the essential oil selectively suppresses the proliferation of A549 lung cancer cells and induces cell death through cell cycle disruption, increased ROS, mitochondrial membrane depolarization, and caspase activation. Overall, studies using *C. verum* and *C. tamala* suggest potential chemopreventive effects through modulation of apoptosis, proliferation, angiogenesis, and related cellular pathways. However, further studies are required to establish the contribution of individual constituents to these effects [86].

### 5.5. Antimicrobial and Antiparasitic

Bacteria can undergo progressive changes in response to environmental stress, and the emergence of strains resistant to almost all currently available antibiotics has become a serious global health threat [87]. In addition to bacteria, parasites are a group of eukaryotic pathogens that include protozoa, helminths, and arthropods that spend part or all of their life cycle in their host organism [88]. Therefore, natural products and their derivatives remain a major source for the discovery and development of drug candidates for the treatment of various human diseases [87].

*C. burmannii* essential oil showed strong antimicrobial activity against skin pathogens, especially Malassezia furfur, with MIC and MFC values of 0.88 and 1.75 mg/mL, respectively, confirming its fungistatic to fungicidal effects. The effect also increased when combined with ketoconazole, as indicated by an FICI of 0.5, suggesting synergism. In addition to inhibiting planktonic growth, treatment at 2× MIC significantly suppressed biofilm formation by up to 85.6%, suggesting its potential to inhibit pathogen colonization and persistence. Morphological analysis using SEM showed surface invagination and pore formation in fungal cells, consistent with increased membrane permeability and leakage of intracellular components (proteins, nucleic acids, and ions). Mechanistically, testing with ergosterol shows that the main target of this essential oil is the cell membrane, as supported by a dose-dependent decrease in ergosterol synthesis and inhibition of squalene epoxidase activity, thereby disrupting membrane stability and ultimately triggering microbial cell death. GC-MS profiling further identified D-borneol as the predominant constituent of CBEO (27.84%), together with α-terpineol, α-pinene, D-limonene, and other monoterpenes, suggesting that the antifungal activity may arise from the combined contribution of multiple volatile constituents rather than a single compound [69].

*C. verum* extract also exhibits antiparasitic activity, as demonstrated by its chemotherapeutic potential against piroplasmal protozoa in vitro and in vivo. Growth inhibition tests showed that acetone and ethyl acetate extracts suppressed the multiplication of *Babesia* spp. and *Theileria equi* in a dose-dependent manner, with IC_50_ values in the range of micrograms per milliliter and a relatively favorable selectivity index against several mammalian cell lines. In addition, microscopic observations revealed changes in parasite morphology after treatment, indicating disruption of the division process and decreased parasite viability during incubation. The consistency of this inhibitory effect was further supported by in vivo testing in mice infected with *Babesia microti*. Administration of the extract at a dose of 150 mg/kg significantly reduced parasitemia. Overall, these results confirm that *C. verum* has the potential to be developed as a candidate for natural-based antipiroplasmic agents, especially as a supporting alternative to the limitations of conventional therapy due to toxicity and drug resistance [89].

### 5.6. Cardioprotective and Neuroprotective

Cardioprotection is the ability of the heart muscle to withstand metabolic stress, including ischemia–reperfusion injury, mediated by endogenous mechanisms. This protective response can be induced through various procedures and compounds, including ischemic preconditioning in the form of a brief episode of ischemia–reperfusion before the occurrence of prolonged stress, postconditioning given after the main injury, and remote preconditioning through ischemia–reperfusion in noncardiac tissue. In addition, cardioprotection can also be triggered by exposure to hypoxia with a certain level of severity, such as a decrease in oxygen tension that is subhypoxic [90].

Neuroprotection is a biological strategy aimed at maintaining the structural and functional integrity of the nervous system, including the brain, spinal cord, and peripheral nerves. This approach helps prevent or limit damage to neurons and other nerve components, thereby maintaining cognitive, sensory, and motor functions. Specifically, neuroprotection focuses on protecting the central nervous system from injury due to acute disorders such as stroke and trauma, as well as progressive neurodegenerative diseases such as dementia, Parkinson’s disease, Alzheimer’s disease, and epilepsy. Herbal medicines and nutraceuticals are seen as potential sources for preventing neurological disorders. This is because phytoconstituents have been reported to modulate the nervous system via specific biological mechanisms in various experimental models [91].

*C. verum* bark extract showed cardioprotective effects in Wistar rats subjected to doxorubicin-induced cardiotoxicity. The doxorubicin group showed increases in biomarkers of myocardial damage, namely cardiac troponin I (cTnI), NT-proBNP, AST, and LDH. Meanwhile, pre-treatment with *C. verum* extract at an optimal dose of 2.0 g/kg significantly reduced these parameters compared with doxorubicin control. This treatment improved the biochemical status of heart tissue by increasing endogenous defense capacity, including GSH, DPx, GR, SOD, and catalase. In addition, it suppressed markers of oxidative damage and inflammation, as indicated by decreases in lipid peroxidation (MDA) and myeloperoxidase (MPO) activity. Histopathologically, the group receiving the extract showed a more preserved myocardial structure with a milder degree of necrosis than the doxorubicin group, indicating that the extract has the potential to protect heart tissue by suppressing oxidative stress and inflammation induced by cardiotoxic agents [92].

*C. bejolghota* bark extract also demonstrated neuroprotective activity against H_2_O_2_-induced oxidative stress in SH-SY5Y neuroblastoma cells. Pre-treatment with the powder extract significantly reduced LDH activity, intracellular ROS levels, and lipid peroxidation while improving neuronal cell viability. At the compound level, cinnamaldehyde has been reported to exhibit neuroprotective effects in independent experimental studies, involving mechanisms such as increased Bcl-2 expression, suppression of Bax expression, preservation of mitochondrial membrane potential, inhibition of cytochrome c release, and reduction in caspase-3 activity. However, the specific contribution of cinnamaldehyde to the neuroprotective activity observed in Cinnamomum extracts remains to be further established [93]. The main pharmacological activities of *Cinnamomum* species and a summary of their experimental evidence are summarized in Table 3.

## 6. Chemometric Analysis in Characterization of Phytochemical Profile and Bioactivity Correlation of *Cinnamomum* spp.

The application of chemometrics on *Cinnamomum* species provides an important contribution to improving the accuracy of phytochemical data interpretation and strengthening the relationship between chemical composition and biological activity [62,102]. In addition, this approach plays a role in the development of metabolite fingerprint-based quality control systems, species authentication, and the determination of compound markers for the standardization of natural products [56,61]. The integration of chemometric analysis with metabolomics and bioactivity approaches is expected to accelerate the identification of active compound candidates, thereby supporting the development of *Cinnamomum* species as a source of standardized, evidence-based phytopharmaceutical raw materials. The application of various chemometric approaches in phytochemical analysis and the correlation of *Cinnamomum* species bioactivity are systematically summarized in Table 4.

Research on *Cinnamomum* has extensively used PCA to evaluate phytochemical profiles, identify chemotypes, and distinguish species based on their chemical and biological characteristics. In a study conducted by Rana et al. (2021) [105], which combined physicochemical properties with chemometric analysis, PCA successfully separated four *Cinnamomum* species: *C. verum*, *C. burmannii*, *C. loureiroi*, and *C. cassia*. The PCA results explained 81.70% of the total variation across three principal components (PC1–PC3), thereby supporting species authentication and the detection of adulteration. PCA analysis also successfully clustered samples of *C. verum* leaf essential oil based on variations in the content of eugenol, benzyl benzoate, (E)-cinnamyl acetate, and (E)-cinnamaldehyde, thereby supporting the identification of chemotypes and the authentication of raw material sources [103]. PCA combined with GC-MS on *C. burmannii* samples revealed the presence of several essential oil chemotypes with different compositions, although the relationship between chemical profiles and chloroplast phylogeny was relatively weak [108]. In *C. cassia*, PCA was used to evaluate germplasm diversity using morphological traits, essential oil content, and single-nucleotide polymorphism (SNP) markers, where leaf length, width, elevation, and oil content were the primary factors contributing to population variation [106]. The application of PCA to UPLC-QTOF-MS data also successfully distinguished the metabolite profiles of *C. tamala* leaf extracts under various supercritical CO_2_ extraction conditions, thereby helping to determine the optimal extraction conditions based on bioactive metabolite content [104]. Furthermore, PCA applied to the essential oil profiles of *C. kanehirae* leaves and related species was able to cluster samples based on chemotypes and distinguish between species and natural hybrids based on their chemical fingerprints [107].

HCA is also widely used in *Cinnamomum* research to evaluate the similarity of phytochemical profiles, group samples based on metabolite composition, and support species authentication. HCA, combined with the HPLC analysis of five major compounds—namely coumarin, cinnamyl alcohol, cinnamic acid, 2-methoxycinnamic acid, and cinnamaldehyde, successfully distinguished young twigs (*Cinnamomi Ramulus*) from bark (*Cinnamomi Cortex*), indicating that these five compounds have the potential to serve as markers for differentiating the two traditional medicinal raw materials [63]. HCA, combined with UPLC-QTOF-MS metabolomics data, also successfully clustered supercritical CO_2_ extracts of *C. tamala* leaves based on extraction conditions, thereby facilitating the selection of optimal conditions that yield the highest levels of fatty acids, fatty acid amides, and cinnamic acid derivatives [104]. Furthermore, HCA was able to cluster GC-MS-based essential oil profiles from several *Cinnamomum* species—namely *C. kanehirae, C. camphora, and C. micrathum*—based on chemotypes, thereby supporting species differentiation, the identification of natural hybrids, and classification based on chemical fingerprints [107]. The HCA approach is widely used in conjunction with PCA to visualize the degree of similarity among samples based on phenolic compound content and biological activity, thereby making the interpretation of metabolomic data easier and more biologically meaningful [109].

PLS and PLS-DA are widely used in *Cinnamomum* research to link phytochemical profiles to biological characteristics and to classify samples based on their chemical fingerprints. PLS-DA successfully linked metabolite profiles to biological activity, thereby facilitating the identification of compounds that contribute most to the activity of the samples [63]. PLS-DA was also able to distinguish two varieties of *C. longepaniculatum* based on their metabolite profiles; this was followed by OPLS-DA using the variable importance in projection (VIP > 1), fold change, and *p*-value parameters, which successfully identified 49 differential metabolites—primarily those involved in biosynthetic pathways, flavonoids, phenylpropanoids, and terpenoids [102]. Furthermore, the combination of chemical composition data, MIR spectroscopy, and chemometric analysis was able to distinguish between genuine and counterfeit cinnamon with a classification accuracy of 94.44% based on chemical composition and 100% based on MIR data, making it a potential rapid method for the authentication and quality control of cinnamon products [110].

Other chemometric approaches used in *Cinnamomum* research include OPLS and OPLS-DA. These analyses can improve discriminatory power and identify the metabolites that contribute most to the differences between groups. OPLS-DA, applied to various *Cinnamomum* species and commercial products using GC-MS, NMR, and UV, successfully distinguished between species and identified coumarin, (E)-methoxycinnamaldehyde, and cinnamic acid as key markers for the authentication and quality control of raw materials [56]. The combination of OPLS-DA with LC-MS metabolomic profiling successfully identified differential metabolites associated with variations in chemical composition among samples, thereby facilitating the identification of biomarker candidates for standardization and evaluation of biological activity [63]. The OPLS-DA approach was also used to link metabolite profiles to the antidiabetic activity of various *C. verum* varieties, thereby helping to identify compounds that contribute to the inhibition of α-glucosidase and α-amylase [114].

In addition to classification-based chemometric methods, statistical analyses such as ANOVA followed by Tukey’s test are widely used to evaluate significant differences in metabolite content and biological activity among species, plant parts, or extraction treatments, thereby enabling the identification of statistically distinct sample groups [114]. Multivariate approaches that integrate phytochemical, physicochemical, and bioactivity data also allow for a more comprehensive interpretation of relationships among variables, thereby improving the accuracy of species authentication, biomarker identification, adulteration detection, and the identification of candidate bioactive compounds in *Cinnamomum* [113,115,116].

Beyond their application in species classification, authentication, and biomarker discovery, chemometric approaches also provide insights into the potential relationship between metabolite patterns and pharmacological properties of *Cinnamomum* species. Several metabolites identified as discriminant markers in chemometric analyses, including cinnamaldehyde, eugenol, cinnamic acid derivatives, and coumarin, have been reported to contribute to various biological activities, such as antioxidant, anti-inflammatory, antimicrobial, and metabolic regulatory effects [5,27,51,69,81]. Therefore, the integration of chemometric profiling with biological activity data may enhance the interpretation of metabolite–activity relationships and support the identification of bioactive compounds responsible for the pharmacological potential of *Cinnamomum* species.

## 7. Toxicological Aspects and Safety Profile of *Cinnamomum* Species

Cinnamon has been reported to have a relatively low toxicity profile when used as a spice or flavoring agent. However, the safety of specific *Cinnamomum* species and preparations should be evaluated considering the species, plant part, extract type, dose, and duration of exposure [73]. The total phenol content of de-oiled *C. longepaniculatum* leaves (DCL-TP) showed a good safety profile in a series of toxicology tests. Acute oral administration of DCL-TP to mice up to a dose of 10 g/kg bw for 14 days did not cause death or toxic symptoms, so the LD_50_ value was stated as >10 g/kg bw. In addition, genotoxicity evaluation using the Ames test showed that DCL-TP was neither mutagenic nor genotoxic at the tested dose range in the mammalian erythrocyte micronucleus test and the spermatocyte chromosome aberration assay. In a 28-day oral toxicity test in Sprague-Dawley (SD) rats with doses up to 2.5 g/kg bw/day, no significant changes were found in feed consumption, body weight, urinalysis, hematological parameters, and blood biochemistry. In addition, macroscopic and histopathological examination of vital organs revealed findings that remained normal compared with controls [49].

Toxicological testing of *C. cassia* extract, administered orally to rats at doses up to 2000 mg/kg bw, showed no mortality or significant toxic effects over 14 days of repeated testing. In a 13-week subchronic test, a high dose of 2000 mg/kg bw was associated with increases in relative liver and kidney weights and total cholesterol; no specific histopathology was observed. Genotoxicity tests (Ames, in vitro, and in vivo micronucleus) also showed negative results, indicating that this extract was neither mutagenic nor clastogenic under the test conditions [117]. Another study reported that *C. camphora* seed kernel oil (CCSKO) demonstrated a high oral safety profile in subchronic toxicity and teratogenicity tests. Administration of CCSKO for 90 days to SD rats at a dose of 1–4 mL/kg bw did not cause mortality or significant clinical changes. In addition, it also does not affect body weight, feed consumption, hematological parameters, blood biochemistry, electrolytes, urinalysis, organ weight, and histopathological features. Teratogenicity tests on pregnant Wistar rats (given on gestational days 6–15) also showed no signs of toxicology in the mother or fetus and no significant increase in external, visceral, or skeletal developmental abnormalities, so that the No Observed Adverse Effect Level (NOAEL) was set at > 4 mL/kg bw [118].

The methanol extract of *C. burmannii* stem bark showed potential biological toxicity in the Brine Shrimp Lethality Test (BSLT), with LC_50_ values ranging from 341.95 ± 32.24 to 826.10 ± 57.79 µg/mL, indicating cytotoxicity at certain concentrations. The LC_50_ values varied across samples from different cultivation locations, confirming that geographical factors contribute to differences in secondary metabolite content and toxicological profiles. All extracts were in the LC_50_ category (<1000 µg/mL) and were therefore classified as toxic under the BSLT classification system. However, the range of values was still at a non-extreme level of toxicity. It was experimentally correlated with the estimated acute oral LD_50_ of > 2500 mg/kg, so that *C. burmannii* stem bark within reasonable consumption limits was considered to have relatively good tolerability. A specific toxicological concern is coumarin, which occurs at relatively higher levels in false cinnamon species such as *C*. *burmannii* and has been associated with hepatotoxicity when consumed excessively, accordingly, a tolerable daily intake of 0.1 mg/kg body weight has been established [119].

## 8. Study Limitations

The main limitation in the literature review of *Cinnamomum* species is the lack of a consistent, standardized framework for linking phytochemical profiles to measurable biological activity. Reported research reviews are based solely on complex extracts or essential oils without adequate determination of marker compounds; the contribution of each compound is not clearly defined. Furthermore, differences in test models across studies, including variations in concentration, exposure time, positive controls, and statistical analysis methods, continue to limit the reproducibility and validity of comparisons of results. Another gap is the lack of information on pharmacokinetics, bioavailability, and stability of bioactive compounds. These aspects are important requirements in assessing the suitability of medicinal plants as therapeutic candidates. However, clinical evidence remains limited, requiring further, more standardized, mechanism-based research to advance the development of applications in the pharmaceutical and healthcare fields.

## 9. Future Perspectives and Research Opportunities

Further research on *Cinnamomum* species should focus on identifying the most promising candidate species and plant parts, and on mapping the key bioactive components most relevant to pharmacological targets. Mechanism-based approaches, integrating phytochemical and pharmacological data, should be strengthened to identify compounds that govern activity and potential synergistic effects within the extract matrix. Furthermore, developing formulations to improve stability and bioavailability is an important strategy to advance potential therapeutic applications. Translational validation of efficacy and safety, particularly through targeted in vivo studies and controlled clinical trials, is a key step toward *Cinnamomum* species becoming a preclinical, evidence-based phytopharmaceutical candidate.

## 10. Conclusions

This review presents a comprehensive overview of the phytochemical content and biological activities of various *Cinnamomum* species from a pharmacological perspective. The results of this study indicate that *Cinnamomum* species are sources of volatile and non-volatile secondary metabolites from various chemical classes associated with multidimensional pharmacological activities, including antioxidant, anti-inflammatory, antidiabetic, antimicrobial/antiparasitic, anticancer/chemopreventive, cardioprotective, and neuroprotective effects. Mechanistically, these effects are generally mediated by regulating oxidative stress, modulating inflammatory mediators, inhibiting enzymatic targets, and intervening in molecular signaling pathways implicated in the pathogenesis of metabolic and degenerative diseases. The application of chemometric analysis makes an important contribution to interpreting the complexity of phytochemical data, identifying marker compounds, and clarifying the relationship between chemical composition and the bioactivity of this plant. Chemometric approaches further support the integration of phytochemical and pharmacological data by revealing associations between specific secondary metabolite profiles and biological responses. Variations in phenylpropanoids, terpenoids, flavonoids, and other phenolic compounds may contribute to differences in antioxidant, antimicrobial, anti-inflammatory, and metabolic activities among *Cinnamomum* species, suggesting their potential roles as chemical markers for bioactivity prediction. However, comparative interpretation across studies is still limited by species heterogeneity, plant differences, variations in extraction methods, and non-uniform experimental designs that affect outcome parameters. Therefore, the development of *Cinnamomum* spp. as a phytopharmaceutical candidate requires the standardization of raw materials and processes, validation of mechanisms through a systematic approach, measurable toxicological evaluation, and confirmation of efficacy through translational studies and controlled clinical trials. With this framework, *Cinnamomum* species has a strong prospect as a source of natural-based therapeutic agent candidates to strengthen innovation in the pharmaceutical and healthcare sectors.

## Figures and Tables

**Table 1:** Major species of *Cinnamomum* used in pharmacological studies.

Species	Common Name	Parts of the Plant Used	Main Pharmacological Activities	Author(s)	Ref.
*Cinnamomum* *verum*	Ceylon cinnamon	Bark, leaves, stems, roots	Antioxidant, anti-inflammatory, antiaging, antidiabetic	Kim et al.; Yan et al.; Mancal et al.	[27,28,29]
*Cinnamomum cassia*	Cassia cinnamon	Bark, leaves	Anti-inflammatory, antioxidant, antidiabetic,	Lee et al.; Lim et al.; Zhou et al.; Cordero-Perez et al.	[30,31,32,33]
*Cinnamomum* *burmannii*	Indonesian cinnamon	Bark, leaves	Antioxidant, antiaging, antidiabetic, anti-inflammatory	Susilowati et al.; Muhammad et al.; Qarani et al.; Rachid et al.; Zhang et al.	[34,35,36,37,38]
*Cinnamomum* *camphora*	Camphor tree	Leaf	Antioxidant, anti-inflammatory	Zhang et al.; Kang et al.; Ling et al.	[39,40,41]
*Cinnamomum* *porrectum*	*Cinnamomum* parthenoxylon	Bark, leaves	Antioxidants	Saetan et al.; Buru et al.	[42,43]
*Cinnamomum tamala*	Indian Cassia	Leaf	Antioxidant, antidiabetic	Kumar et al.	[44]
*Cinnamomum* *subavenium*	*Cinnamomum* randaiense hayata	Leaf	Antioxidant, antimicrobial, anticancer	Ho et al.; Liu et al.	[45,46]
*Cinnamomum* *chartophyllum*	-	Aerial parts	Antioxidants	Shen et al.; Zhou et al.	[47,48]
*Cinnamomum* *longipaniculatum*	-	Leaf	Antioxidant, antidiabetic	Li et al.; Liu et al.	[49,50]
*Cinnamomum* *malabatrum*	Malabar cinnamon	Leaf	Antioxidant, antidiabetic	Kuttithodi et al.	[51]
*Cinnamomum* *japonicum*	*Cinnamomum* pedunculatum Nees	Branches, leaves	Antioxidant, anti-inflammatory	Kim et al.; Kim et al.	[52,53]
*Cinnamomum* *loureiroi*	Saigon cinnamon	Bark, leaves	Antidiabetic, antioxidant, antimicrobial	Mancak et al.; Tanapichatsakul et al.	[29,54]

**Table 2:** Phytochemical profile of *Cinnamomum* species based on compound group, chemical class, plant part, and identification method.

Compound	Chemistry Class	Species (*Cinnamomum*)	Plant Parts	Identification Method	Ref.
Eugenol	Phenylpropanoid	*C. verum;* *C. japonicum.*	Leaves; Branches; bark	GC-MS (tentative); HPLC-DAD (confirmed)	[52,65,66,67]
Benzyl benzoate	Aromatic esters	*C. verum*	Leaf	GC-MS (tentative)	[65,66,67]
Trans caryophyllene	Sesquiterpene	*C. verum*	Leaf	GC-MS (tentative)	[65,66]
Acetyl eugenol	Phenylpropanoid esters	*C. verum*	Leaf	GC-MS (tentative)	[65,66]
Linalool	Monoterpenoid alcohol	*C. verum*	Leaves; bark	GC-MS (tentative)	[65,66,67]
Trans- Cinnamaldehyde	Phenylpropanoid	*C. verum*	Leaf	GC-MS (tentative)	[65]
Cinnamyl acetic acid ester	Phenylpropanoid esters	*C. verum*	Leaf	GC-MS (tentative)	[65]
α-Pinene	Monoterpenes	*C. verum*	bark	GC-MS (tentative)	[67,68,69]
Camphene	Monoterpenes	*C. verum*	bark	GC-MS (tentative)	[68]
Benzaldehyde	Aromatic aldehyde	*C. verum*	bark	GC-MS (tentative)	[68]
3-Phenylpropanal	Phenylpropanoid aldehyde	*C. verum*	bark	GC-MS (tentative)	[68]
Isoborneol	Monoterpenoid alcohol	*C. verum*	bark	GC-MS (tentative)	[68]
Cyclosativene	Sesquiterpenes	*C. verum*	bark	GC-MS (tentative)	[68]
Cinnamaldehyde dimethyl acetal	Acetal	*C. verum*	bark	GC-MS (tentative)	[67,68]
Isosativene	Sesquiterpenes	*C. verum*	bark	GC-MS (tentative)	[68]
(Z)-α-Bisabolene	Sesquiterpenes	*C. verum*	bark	GC-MS (tentative)	[68]
Aromadendrene	Sesquiterpenes	*C. verum*	bark	GC-MS (tentative)	[68]
Cinnamaldehyde	Phenylpropanoid	*C. cassia;* *C. burmannii;* *C. verum*	Bark; leaves	GC-MS (tentative)	[36,67,68,70]
δ-cadinene	Sesquiterpenes	*C. cassia; C. verum*	bark	GC-MS (tentative)	[68,70]
α-copaene	Sesquiterpenes	*C. cassia; C. verum*	bark	GC-MS (tentative)	[68,70]
α-murolene	Sesquiterpenes	*C. cassia; C. verum*	bark	GC-MS (tentative)	[68,70]
γ-cadinene	Sesquiterpenes	*C. cassia; C. verum*	bark	GC-MS (tentative)	[66,70]
Endo-borneol	Monoterpenoid alcohol	*C. burmannii*	Leaves; branches	GC-MS (tentative)	[38,69]
Linalyl propanoate	Monoterpenoid esters	*C. burmannii*	Leaf	GC-MS (tentative)	[38]
Isobornyl acetate	Monoterpenoid esters	*C. burmannii*	Leaves; branches	GC-MS (tentative)	[38,69]
(+)-2-Bornanone	Monoterpenoid ketone	*C. burmannii*	Leaf	GC-MS (tentative)	[38]
Eucalyptol	Monoterpenoid oxide	*C. burmannii*	Leaf	GC-MS (tentative)	[38]
α-Terpineol	Monoterpenoid alcohol	*C. burmannii; C. verum*	Leaves; branches; bark	GC-MS (tentative)	[38,67,69]
Caryophyllene	Sesquiterpenes	*C. burmannii;* *C. verum*	Leaves; bark	GC-MS (tentative)	[38,67]
Terpinen-4-ol	Monoterpenoid alcohol	*C. burmannii*	Leaf	GC-MS (tentative)	[38]
5-hydroxymethylfurfural (5-HMF)	Furan aldehyde	*C. burmannii*	Leaf	GC-MS (tentative)	[36]
Benzene, 1-methyl-3-(1-methylethyl)	Aromatic hydrocarbons	*C. burmannii*	Leaves; branches	GC-MS (tentative)	[69]
D-Limonene	Monoterpenes	*C. burmannii; C. verum*	Leaves; branches; bark	GC-MS (tentative)	[67,69]
Geranial	Monoterpenoid aldehyde	*C. camphora*	Leaf	GC-MS (tentative)	[41]
Neral	Monoterpenoid aldehyde	*C. camphora*	Leaf	GC-MS (tentative)	[41]
Safrole	Phenylpropanoid	*C. porrectum*	bark	GC-MS (tentative)	[71]
Elemicin	Phenylpropanoid	*C. porrectum*	bark	GC-MS (tentative)	[71]
Methyl eugenol	Phenylpropanoid	*C. porrectum*	bark	GC-MS (tentative)	[71]
Cinnamyl acetate	Phenylpropanoid esters	*C. japonicum; C. verum*	Branches; bark	HPLC-DAD (confirmed); GC-MS (tentative)	[52,67]
Cinnamyl alcohol	Phenylpropanoid alcohol	*C. japonicum; C. burmannii*	Branches; leaves	HPLC-DAD (confirmed); GC-MS (tentative)	[36,52]
Epicatechin	Flavonoid	*C. japonicum*	Branch	HPLC-DAD (confirmed)	[52]
Epigallocatechin gallate	Flavonoid	*C. japonicum*	Branch	HPLC-DAD (confirmed)	[52]
Trans-cinnamic acid	Phenylpropanoid	*C. japonicum*	Branch	HPLC-DAD (confirmed)	[52]
Quercetin	Flavonoid	*C. japonicum*	Branch	HPLC-DAD (confirmed)	[52]
2-amino-5-guanidino-pentanoic acid	Amino acids	*C. burmannii*	Leaf	GC-MS (tentative)	[36]
2,3-dihydro-3,5-dihydroxy-6-methyl-4H-pyran-4-one	Oxygen-containing heterocyclic compound	*C. burmannii*	Leaf	GC-MS (tentative)	[36]
Azelaic acid	Dicarboxylic acid	*C. verum*	bark	UPLC-QTOF/MS (tentative)	[27]
Cinnamic acid	Phenylpropanoid	*C. verum*	bark	UPLC-QTOF/MS (tentative)	[27]
Coumarin	Coumarin	*C. verum;* *C. burmannii*	bark	UPLC-QTOF/MS (tentative); GC-MS (tentative)	[27,36]
Linoleic acid derivatives	Polyunsaturated fatty acids derivatives	*C. verum*	bark	UPLC-QTOF /MS (tentative)	[27]
α-linoleic acid derivatives	Polyunsaturated fatty acids derivatives	*C. verum*	bark	UPLC-QTOF/MS (tentative)	[27]

**Table 3:** Pharmacological activity, experimental model, parameters assessed, and main outcomes.

Activity	Sample Type	Experimental Model	Parameters Assessed	Main Outcome	Ref.
Antioxidants	Extract/ essential oil, *Cinnamomum* fraction (leaves, bark, branches, including after simulated digestion)	In vitro (DPPH, ABTS, reducing power), cell model (ROS assay), and digestion simulation + cellular evaluation	IC_50_ DPPH/ABTS, reduction capacity, intracellular ROS, total phenolics, total phenolics/ flavonoids	In general, it shows significant antioxidant activity (low IC_50_, reduced ROS) associated with phenolics/ flavonoids, although this may decrease after digestion.	[5,27,52,53,78]
Anti- inflammatory	Extract, essential oil, and active components of *Cinnamomum* (leaves, bark, including after simulated digestion)	In vitro inflammation model: RAW 264.7 + LPS; THP-1 + PMA; intestinal/ barrier cell model after simulated digestion	NO (Griess), iNOS/COX-2 expression, proinflammatory cytokines (IL-1β, IL-8, CCL5), PGE2, ROS, activation of the AP-1/NF-κB pathway and related proteins (e.g., PKCδ/JNK, NOX2	In general, it shows significant anti- inflammatory activity through suppression of NO/cytokines, reduction of iNOS/COX-2, and inhibition of the AP-1/NF-κB pathway.	[13,21,27,77]
Antidiabetic	Extract and formulation	In vitro (α-glucosidase/α-amylase, glucose uptake); in vivo (diabetic model)	IC _50_ enzymes, glucose/OGTT, insulin sensitivity	Antidiabetic potential through enzyme inhibition and improvement of glycemic control.	[80,81,82,93,94]
Anticancer and chemopreventive	Extracts, essential oils of bark/leaves, EO-based mixtures, and compounds/ formulated products	In vitro: cytotoxicity assays on various cancer cell lines (e.g., breast, colon, lung) ± mechanism analysis (apoptosis & cell cycle); in vivo: tumor/ chemoprevention models (e.g., breast cancer)	Viability/IC_50_ and selectivity towards normal cells, apoptosis (Annexin V/PI, caspase-3/7, Bax/Bcl-2), proliferation (Ki67), angiogenesis (VEGF), cell cycle (Cyclin D, p-AKT), and indicators of oxidative stress related to tumor progression	Anticancer potential (selective cytotoxicity, antiproliferation, apoptosis induction, cell cycle arrest) and chemopreventive (suppression of tumor progression through modulation of proliferation/ angiogenesis markers and oxidative stress) with effectiveness influenced by sample type and formulation approach.	[16,23,67,85,95,96,97]
Antimicrobial/ antiparasitic	Essential oils and extracts (bark/leaves/branches)	Antibacterial (in vitro): disk diffusion and microdilution (MIC/MBC); biofilm assay; antiparasitic (in vitro & in vivo): protozoa/ piroplasm viability assay & mouse test; evaluation of potential synergy with antibiotics/fungi	Zone of inhibition, MIC/MBC, MIC/MMC, synergy index (FICI), antiparasitic IC_50_ & selectivity, indicator of infection/ parasitemia reduction (in vivo)	Demonstrates significant antibacterial and antiparasitic activity including potential synergy with antimicrobial drugs with the general outcome of reducing the growth of microorganisms/ parasites at effective concentrations.	[69,88,98,99,100]
Cardioprotective/ neuroprotective	Bark extract and other bioactive components	Neuroprotective (in vitro): SH-SY5Y cells + oxidative stress induction (H_2_O_2_); Cardioprotective (in vivo): Wistar rats + doxorubicin cardiotoxicity	ROS, LDH, lipid peroxidation (TBARS), cardiac biomarkers (cTnI, NT-proBNP, AST/LDH), TAC, GSH, SOD, CAT, GPx/GR, MPO, histopathology	In general, it shows neuroprotective effects (decreased ROS/LDH, and lipid peroxidation) and cardioprotective effects (decreased cardiac injury biomarkers and increased tissue antioxidant status).	[91,101]

**Table 4:** Chemometrics analysis for the characterization of phytochemical profiles and the correlation analysis of the bioactivity of *Cinnamomum* species.

Chemometric Techniques	Purpose of Analysis	Data Used	Main Output	*Cinnamomum* Studies	Ref.
PCA	Exploration of data structure and variation; sample grouping	Metabolite and volatile profiles (GC-MS/LC-MS); physico-functional properties; genetic data and phenotypic characters; phytochemical and bioactivity data	Score plot; loading plot; sample clustering pattern; PC contribution to variation	Species discrimination and origin; genetic structure and population differentiation	[103,104,105,106,107,108,109]
HCA/AHC	Similarity-based clustering	Metabolite profile (LC-MS/GC-MS); phytochemical and bioactivity data	Dendrogram and heatmap	Identification of compositional similarities; sample classification	[63,104,107,108,109]
PLS/ PLS-DA	Relationship between composition and biological activity; sample classification based on chemical and spectral profiles	IC_50+_ phytochemical data; peak area of LC-MS metabolites	VIP score; regression; classification model; accuracy; sensitivity; specificity	Identification of bioactive markers; authentication and quality control of materials	[63,110,111,112]
OPLS/ OPLS-DA	Selection of important variables; directional discrimination between sample groups	Metabolomics; peak area of LC-MS metabolites; GC-MS data; UV; NMR	Candidate biomarkers; differential metabolites; VIP score; classification model	Determination of lead compounds; sample differentiation; key metabolites	[56,63,112]
ANOVA-Tukey	Evaluation of character differences between samples	Quantitative phytochemical data; physicochemical; bioactivity	Significance model and group separation; pattern of significant differences between treatments	Determination of the most effective treatment and composition	[51,113,114]
Multivariate approach	Integration of chemical and sensory parameters	Data is multidominant	Classification model; sample cluster	Species authentication and adulteration detection; species differentiation and sample identification	[115,116]

## Data Availability

Dataset available on request from the authors.
